# Monte Carlo determination of radiation‐induced cancer risks for prostate patients undergoing intensity‐modulated radiation therapy

**DOI:** 10.1120/jacmp.v8i4.2685

**Published:** 2007-09-17

**Authors:** Sotirios Stathakis, Jinsheng Li, Charlie C.M. Ma

**Affiliations:** ^1^ Cancer Therapy and Research Center San Antonio Texas; ^2^ Department of Radiation Oncology Fox Chase Cancer Center Philadelphia Pennsylvania U.S.A.

**Keywords:** IMRT, secondary malignancies, prostate radiation therapy

## Abstract

The application of intensity‐modulated radiation therapy (IMRT) has enabled the delivery of high doses to the target volume while sparing the surrounding normal tissues. The drawbacks of intensity modulation, as implemented using a computer‐controlled multileaf collimator (MLC), are the larger number of monitor units (MUs) and longer beam‐on time as compared with conventional radiotherapy. Additionally, IMRT uses more beam directions—typically 5 – 9 for prostate treatment—to achieve highly conformal dose and normal‐tissue sparing. In the present work, we study radiation‐induced cancer risks attributable to IMRT delivery using MLC for prostate patients.

Whole‐body computed tomography scans were used in our study to calculate (according to report no. 116 from the National Council on Radiation Protection and Measurements) the effective dose equivalent received by individual organs. We used EGS4 and MCSIM to compute the dose for IMRT and three‐dimensional conformal radiotherapy. The effects of collimator rotation, distance from the treatment field, and scatter and leakage contribution to the whole‐body dose were investigated. We calculated the whole‐body dose equivalent to estimate the increase in the risk of secondary malignancies.

Our results showed an overall doubling in the risk of secondary malignancies from the application of IMRT as compared with conventional radiotherapy. This increase in the risk of secondary malignancies is not necessarily related to a relative increase in MUs. The whole‐body dose equivalent was also affected by collimator rotation, field size, and the energy of the photon beam. Smaller field sizes of low‐energy photon beams (that is, 6 MV) with the MLC axis along the lateral axis of the patient resulted in the lowest whole‐body dose. Our results can be used to evaluate the risk of secondary malignancies for prostate IMRT patients.

PACS: 87.53.wz, 87.53.‐j

## I. INTRODUCTION

Radiation therapy has long been recognized as an effective treatment for the management of clinically localized prostate cancer. Recently, improvements in treatment outcomes have been clearly demonstrated through dose escalation studies for prostate radiotherapy. The state‐of‐the‐art techniques that facilitate dose escalation are three‐dimensional conformal radiotherapy (3D‐CRT) and, more recently, intensity‐modulated radiotherapy (IMRT). Advanced radiotherapy treatments with IMRT can deliver dose distributions that are more conformal to the tumor targets and that simultaneously minimize radiation damage to the surrounding normal tissues.^(^ ^1^ ^–^ ^7^ ^)^

Overall, IMRT provides increased local tumor control and lower toxicities to nearby critical organs. However, the process of intensity modulation requires more monitor units (MUs) than does conventional radiotherapy or 3D‐CRT. The increase in MUs inevitably leads to an increase in the leakage dose and results in a higher dose to the rest of the patient's body. Depending on the design of the accelerator's multileaf collimator (MLC) and the treatment optimization system used, a usual prostate IMRT treatment may consist of 50 – 100 MLC field segments that may take 3 – 10 times the MUs of a comparable 3D‐CRT or conventional prostate treatment.^(^ ^8^ ^,^ ^9^ ^)^ Compared with conventional treatments, IMRT uses more beam directions (typically 5 – 9) to achieve optimal dose conformity to the target volume while reducing the dose to the surrounding critical structures. Altogether, IMRT treatment may substantially increase the normal‐tissue volume receiving low‐dose radiation over the dose seen in conventional and 3D‐CRT treatment.

In general, radiotherapy has been shown to be associated with a very small, but statistically significant, increase in the risk of secondary malignancies.^(10)^ This increase is more profound in long‐term cancer survivors. It has been reported that secondary lung malignancies increased by 4% – 6% after prostate radiotherapy as compared with prostate surgery, and that the increase rose to as much as 15% for long‐term survivors,^(10)^ although other factors unrelated to radiation (such as smoking) might have contributed. In another study for radiation therapy of the cervix,^(11)^ the risk of secondary malignancies in a wide range of organs was investigated, and higher doses (in the order of several grays) were reported to increase the risk of stomach cancer and leukemia. Movsas et al.^(12)^ observed that 5.7% of patients treated with radiation developed secondary tumors. Dorr and Herrmann^(13)^ showed that most secondary tumors occur in the penumbra region, where the dose is ≤6 Gy, within 11 – 16 years after the initial treatment.

Although the above‐mentioned reports are for conventional and 3D‐CRT treatments, Followill et al.^(8)^ estimated that the percentage likelihood of fatal secondary cancers attributable to a prescribed dose of 70 Gy can be as high as 4.5% for IMRT with 18 MV photon beams and up to 8.4% for 25 MV photon beams.

The dose–response relationship is uncertain in the context of radiotherapy in which a small volume receives a high dose, sometimes 70 Gy or more, and a larger volume receives a considerably lower dose. The low doses can be a result of exposure to only some of the treatment fields or exposure to only leakage radiation from the accelerator's head. Three probable scenarios, all supported by published data, attempt to explain the relationship between the dose received and the risk of secondary malignancies:

- First, from animal studies,^(14)^ the risk of second cancers is expected to fall off at higher doses because of cell killing(dead cells cannot give rise to malignancies).
- Second, based on data from human studies, the risk for development of solid tumors has been observed to level off at 4 – 8 Gy, but not to decline thereafter.^(15)^
- Third, women who have been treated for cervical cancer have an increased risk of developing leukemia. In this situation, the risk increases with the dose up to 4 Gy and then decreases at higher doses.^(^ ^16^ ^–^ ^18^ ^)^

Although it is generally accepted that IMRT improves local tumor control and reduces toxicity to nearby critical structures, questions have been raised concerning whether the widespread use of IMRT could lead to an increase in radiation‐induced carcinomas because a higher volume of normal tissue is being exposed to low‐dose radiation.^(^ ^19^ ^,^ ^20^ ^)^ Although clinical data are still sparse on this subject, we feel that this topic is very important, especially because many community hospitals are implementing IMRT for prostate treatment.

The goal of the present study was to evaluate the overall benefit–risk ratio of IMRT and existing treatment techniques, to calculate the dose received in nearby critical organs and in organs at greater distances, and to compute the whole‐body dose equivalent and the risk of radiation‐induced malignancies resulting from low doses to the rest of the patient's body. We therefore studied the patient scatter dose from the target volume to the organs at risk, the effect of leakage from the linear accelerator (LINAC) head, and the effect of the energy to the whole‐body dose equivalent. We then calculated the relative increase in the risk of secondary cancers attributable to the application of IMRT by taking into account all of the above effects. It is understood that the physical and biologic models and parameters—for example, isodose, dose–volume histogram (DVH), tumor control probability (TCP), normal‐tissue complication probability (NTCP), integral dose, whole‐body dose equivalent, and so on—currently used for plan evaluation and risk analysis are approximate and that their absolute values can be very uncertain. For example, it is difficult to determine the whole‐body dose equivalent for prostate radiotherapy patients because, as a group, these patients are different from a population averaged over a wide range of age, sex, and exposure levels, as considered by the International Commission on Radiological Protection (ICRP)^(21)^ and the National Council on Radiation Protection and Measurements (NCRP)^(22)^. However, because the same parameters and the same patients are being used to evaluate different treatment techniques, only relative changes in the parameters are of significance, and those relative changes are sufficient for this evaluation. Our results are not intended to be used for the prediction of absolute survival and toxicities, but rather to provide useful benefit–risk information for prostate IMRT planning and treatment design so as to promote best utilization of this advanced technology. Our results will also facilitate verification and modification of the theories and models used for tumor control, toxicity, and risk analysis once such clinical data become readily available.

## II. MATERIALS AND METHODS

A fundamental requirement for the benefit–risk calculation is precise knowledge of the dose distribution in the patient. Because this study is based on retrospective patient data, it was not possible to perform measurements to obtain distributions. On the other hand, it is difficult, if not impossible, to accurately measure dose in various organs for every patient whether in retrospective or prospective studies. The Monte Carlo method provides a perfect solution to this problem. Monte Carlo simulations not only can predict dose distributions in heterogeneous patient anatomy, but also can accurately take into account LINAC leakage and patient scatter dose, which are not available in the patient treatment plans and cannot be calculated using any existing commercial treatment planning systems. Furthermore, re‐calculation of the patient dose distribution using different treatment techniques also allows for plan comparison and benefit–risk analysis on an equal basis.

### A. LINAC simulations and source beam models

In the present work, we used the EGS4/BEAM^(23)^ and EGS4/MCSIM^(24)^ Monte Carlo codes for the LINAC simulations and patient dose calculations. The EGS4/BEAM system was developed for the simulation of radiotherapy beams from clinical accelerators.^(25)^ During an accelerator simulation, a phase‐space file is generated that stores information about all the particles exiting the accelerator head, including particle energy, position, direction, and a tag to record particle history (where it has been, and where it was created or has interacted). The geometry and the materials used in the simulation were based on the specifications of the LINAC treatment head as provided by the manufacturer. The cutoff energies used for the simulations were ECUT=700 keV for electrons and PCUT=10 keV for photons. The energy thresholds for d ray production and for bremsstrahlung production were 700 keV and 10 keV respectively. The maximum fractional energy loss per electron step was set to 0.01, and the default parameters were chosen for the parameter reduced electron‐step transport algorithm.^(26)^ Excellent agreement (better than 2%) has been achieved between measurements and the Monte Carlo dose distributions calculated using simulated phase‐space data.^(^ ^23^ ^,^ ^27^ ^)^ Because each phase‐space file requires hundreds of megabytes of disk space, it is necessary to characterize the phase‐space data for widespread clinical applications when hundreds of beam settings may be required. Beam characterization studies show that using simplified beam models can dramatically reduce the storage requirement and increase the efficiency of the accelerator simulation.^(28)^

Source models (SMs) for the Siemens Primus LINACs (Siemens Medical Solutions, Concord, CA) with nominal photon energies of 6, 10, and 18 MV have been constructed from respective phase space files. Our multiple SM consists of an extended annular source for the target, a planar ring source for the primary collimator, and a planar annular source for the flattening filter.^(^ ^28^ ^,^ ^29^ ^)^ The geometry and spatial positions of each source in the SM follow the manufacturers’ specifications and are adjusted to yield the best match in the dose distributions between the original phase space and the reconstructed phase space from our multiple SM. The SMs were used in EGS4/MCSIM, and the dose in water was calculated and compared with ion chamber measurements in a water phantom. The agreement was within 1%.^(30)^ To calculate more accurately the dose to organs outside the treatment field and at distances further away from the lateral extent of the LINAC's largest field size, the source dimensions were extended to simulate the leakage from the LINAC head. The accuracy of the extended SMs was tested with ion chamber (with a buildup cap) measurements in air.

### B. Dose calculations

The EGS4/MCSIM code system can accurately calculate patient dose distributions by simulating the accelerator head leakage, MLC leaf leakage and scatter, and the effect of beam modifiers such as collimator jaws, wedges, and blocks. It also runs 10 – 30 times faster than other widely available general‐purpose Monte Carlo codes.^(24)^

The EGS4/MCSIM code was used in this work because of its extended functionality. It is capable of calculating the dose to the patient given the intensity map or the Radiation Therapy Plan (RTP) file from the Corvus treatment system (Nomos Corp, Sewickley, PA), which includes patient setup parameters, and beam and leaf‐sequence information. The RTP files generated by our inverse treatment planning system for a prostate case were used as input for EGS4/MCSIM. For comparison purposes, the same RTP files were simulated using all three SMs. To investigate the effect of collimator angle, the RTP files were edited so that the collimator was rotated by 90 degrees. The comparison of the two techniques used RTP files for a conventional four‐beam box technique. Based on the dose per incident particle, as derived from calculations using calibration conditions, EGS4/MCSIM calculates the absolute dose to the patient. Moreover, if contours exist in the patient geometry [from computed tomography (CT) images], we are able to calculate the dose to each organ and plot DVHs for all contoured organs.

**Figure 1:**
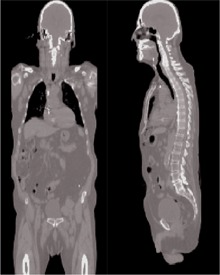
Whole‐body male phantom created from patient computed tomography data for use in Monte Carlo calculations.

For this work, we were interested not only in the dose to the target, but also in the dose outside the target volume, to proximal and distal organs. A phantom created from a patient's whole‐body CT slices (Fig. 1) was used. The phantom describes the patient anatomy, is in rectilinear coordinate system, and is composed of voxels of dimensions 4×4×4 mm. The density of each voxel in the phantom is derived from the respective CT information. The contours for all major organs, mainly the organs indicated by NCRP report no. 116, were outlined so that they could be used for our calculations.

### C. Phantom scatter and head leakage considerations

The dose to the risk organs outside the treatment volume has two components. The first component relates to the scatter radiation from the patient, which is more or less the same for the same target volume and prescription dose. Theoretically, the dose decreases as the target dose distribution becomes more conformal. The second component relates to leakage radiation from the accelerator. Clinical LINACs are designed to have less than 0.1% head leakage (defined as the ratio of the detector readings at any point outside the largest treatment field to that at the center of a 10×10−cm open field, with a 1‐m source‐to‐detector distance).

We used a water phantom to conduct a Monte Carlo study investigating the effect of the scatter and leakage radiation for field sizes ranging from 5×5 cm to 20×20 cm and for all energies under investigation. First, the calculations were performed in the water phantom, and the dose distributions were computed. To separate the phantom scatter from the leakage radiation, we replaced the mass density of a slab in our water phantom to $2000{g/\text{cm}}^{3}$ to ensure that the phantom scatter would not penetrate to the other side of the slab, and we then repeated the calculations as before. Moreover, we investigated the effect of collimator rotation on the whole‐body effective dose. A similar approach was taken to investigate the effects of the scatter and leakage radiation in patient anatomy.

### D. Risk of secondary malignancies

From the dose distributions calculated in the patient, we computed the dose to the outlined organs. For each organ, MCSIM is able to calculate the minimum, maximum, and average doses, together with the volume and the average density. Having all of this data available, the average dose for each organ can then be used to calculate the equivalent dose. The risk of radiation‐induced malignancies is estimated from the difference in the whole‐body effective dose between IMRT and conventional radiotherapy using the recommendations from NCRP report no. 116 for weighting factors for each organ (Table 1). “Re‐weighting” of risk estimates for the prostate patients are applied in the present case, because the population is generally older. It has been reported that the risk estimate for radiotherapy patients should be about 2% per sievert^(31)^ or 1% per sievert for elderly (prostate) patients.

**Table 1:** Risk coefficients for each organ according to the National Council on Radiation Protection and Measurements report no. 116^(22)^

1	Gonads	0.20
2	Colon	0.12
3	Red bone marrow	0.12
4	Lung	0.12
5	Stomach	0.12
6	Bladder	0.05
7	Breast	0.05
8	Liver	0.05
9	Esophagus	0.05
10	Thyroid	0.05
11	Skin	0.01
12	Bone surface	0.01
13	Remainder	0.05

Also, as mentioned earlier, because the dose–response relationship is not well established for organs receiving high doses of radiation, we calculated the whole‐body dose equivalent—and hence the risk of secondary malignancies—using three methods according to the doses received by the proximal organs (bladder and rectum). In method 1, we calculated the whole‐body dose equivalent without any dose restrictions on the nearby organs. The actual dose received was used in the calculations. In method 2, we calculated the whole‐body dose equivalent using a threshold of 4 Gy for the nearby organs, assuming that risk of solid tumors increases up to 4 Gy, but then levels off and does not decline. And in method 3, we did not take into account the dose delivered to the proximal organs, assuming that the risk of secondary cancers falls off at higher doses because of cell killing (dead cells cannot give rise to malignancies).

## III. RESULTS

### A. LINAC verification and head leakage

**Figure 2:**
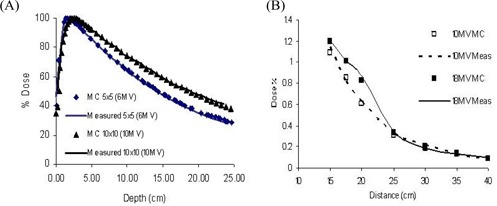
Monte Carlo (MC) linear accelerator verification: (A) Percent depth dose for 5×5−cm and 10×10−cm 10‐MV fields, and (B) point measurements at distances away from the beam central axis.

Monte Carlo simulations of the Siemens Primus 6‐MV, 10‐MV, and 18‐MV beams were verified by comparing the MCSIM dose calculations with measurements for various fields. The calculated percent depth dose curves and dose profiles at various depths for several fields ranging from 5×5 cm to 40×40 cm were compared against measurements. The agreement between measured and calculated values was within 1% or 0.1 cm [Fig. 2(A)].

The verification of our SMs was performed with measurements in air using an ion chamber with a buildup cap. Ion chamber measurements were performed for both axes and extended to 80 cm away from the central axis on the plane perpendicular to the central axis. Measurements in water at 10 cm depth were also made at the same location and used for the SM verification. These measurements were performed for all energies studied [(Figure 2B)].

### B. Studies in water phantom

**Figure 3:**
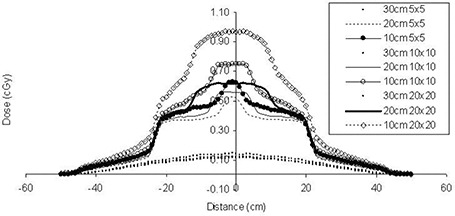
Dose profiles for the 10‐MV photon beam at various distances from the defined field edge of the multileaf collimator. Field size ranges from 5×5 cm to 20×20 cm.

The dose distributions in water were calculated for fields ranging from 5×5 cm to 20×20 cm. The MUs were kept constant (100 MUs) so as to study the effect of leakage radiation attributable to energy and field size (Fig. 3).

### C. Field size dependence

The doses at distances away from the central axis depend on field size. For the same distance from the MLC‐defined field edge, the dose increases with field size. This increase is in the order of 70% from 5×5 cm to 20×20 cm. For the same fields, the respective increase along the axis of the jaw movement is much less—namely, about 20%. Independent of the field size, the dose drops off with distance from the central axis.

As distance from the field edge increases, it is clear that the scatter contribution of the larger fields is similar to those of the smaller ones. For distances closer to the beam edge, the doses for the larger fields are higher. The same characteristics are observed at the sides, where the fields are defined by the jaws. In these cases, the doses are not as high, because transmission through the jaws and the MLC reduces the dose received.

### D. Leakage separation

In our Monte Carlo simulations, the LINAC head leakage was calculated by replacing the density of a slab of the phantom 5 cm from the field edge with a relative density of $2000{g/\text{cm}}^{3}$. Such a slab will stop all the electrons and photons generated in the phantom for the given fields from reaching the other part of the phantom. Therefore, only particles outside the field were scored at distances after the high density slab. These particles will deliver dose that is representative of LINAC head leakage. This leakage was found to be constant, independent of the field size.

The leakage dose attributable to jaws and MLC transmission remains the same for all energies. For points that are far from the central axis and “protected” by the LINAC's head shielding, the dose resulting from leakage is lower and within the manufacturer's specifications.

### E. Scatter contribution

Because we are able to calculate the dose attributable to leakage radiation, the scatter component of the dose at locations outside the field can also be derived. The scatter dose decreases with increasing distance from the central axis, and at the same time, it increases with increasing field size. This field size dependence is attributed to the fact that larger fields generate more scatter radiation that can be carried outside the field. From our calculations, we see that the scatter component of the dose is higher for lower energies than for higher energies. This phenomenon can be attributed to the more forward direction of penetration as the energy increases. The scatter component for the 6‐MV beam is generally higher than that for the 10‐MV and 18‐MV beams. When the distance from the central axis becomes large, the scatter contribution for all energies becomes indistinguishable and is comparable to the leakage component.

### F. IMRT calculations in a water phantom

To study the effect of IMRT treatments in homogeneous geometry, we first used a parallelepiped phantom of dimensions 20×20×50 cm. We placed the isocenter in the phantom, together with its central axis, at a depth of 10 cm. Two treatments were simulated, one representing a four‐beam box technique using four 7×7−cm fields at 0 degrees, 90 degrees, 180 degrees, and 270 degrees, and another representing an IMRT with 7 beams. The dose to the isocenter was 180 cGy for both cases, and different numbers of MUs were used in each case to deliver the prescribed dose.

**Figure 4:**
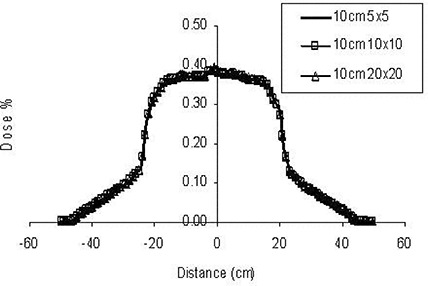
Leakage‐only dose attributable to 6‐MV fields ranging from 5×5 cm to 20×20 cm at 10 cm from the field edge defined by the jaws.

For this study, we also calculated the dose distributions for the same fields, but rotated the collimator by 90 degrees for each field (both for the 7×7 cm fields and for the IMRT fields), so that we could investigate the impact of MLC leakage on the dose. From Fig. 4, we notice that the IMRT treatment increases the dose at distances away from the target and outside the treatment volume. In Fig. 4, the dose profiles from the isocenter are shown at that plane. The collimator setting does not contribute significantly to the whole‐body dose equivalent in the case of the four‐beam technique, but an increase in the whole‐body dose equivalent occurs for the IMRT case with 90 degrees of collimator rotation. This increase could be explained by the increased number of MUs for IMRT, leading to more leakage. From this study, the relative increase in the dose attributable to IMRT can be seen to be explained by the increase in the modulation scaling factor (MSF).^(19)^ The tripling of values from the four‐beam box to IMRT is equal to the MSF for the present study, which was calculated to be 3.5.

### G. Equivalent effective dose and prediction of secondary malignancies

The prescription used for all simulations was 72 Gy to the planning target volume. The whole‐body dose equivalent was calculated based on that prescription and on Table 1.

From the calculated dose distributions, it is evident that, as compared with four‐beam box treatment, application of IMRT increases the whole‐body dose equivalent, and hence the risk of secondary malignancies (Table 2). The risk also increases with increasing energy. Given the assumptions made for the three models described in Materials and Methods, the computed risk seems to increase significantly when the dose for the bladder is included in the calculations (method 1). On the other hand, exclusion of the proximal organs from the calculations of the risk (method 3) reduces the risk of secondary malignancies. We believe that the best case is the use of a 4‐Gy cutoff for the proximal organs (method 2), because at dose levels greater than 4 Gy, radiation may have more cell‐killing effect than cancer‐induction effect.

**Table 2:** Whole‐body dose equivalent (cGy) and estimated percentage (%) likelihood for secondary cancer from a total 72 Gy using the four‐beam box (FBB) technique and intensity‐modulated radiation therapy (IMRT)

	6 MV		10 MV		18 MV	
Method^a^	FBB	IMRT	FBB	IMRT	FBB	IMRT
1	120	177	171	209	172	202
	(2.40)	(3.55)	(3.43)	(4.19)	(3.54)	(4.05)
2	56	133	86	160	86	153
	(1.13)	(2.65)	(1.72)	(3.19)	(1.73)	(3.07)
3	47	123	77	151	78	145
	(0.93)	(2.46)	(1.54)	(3.02)	(1.56)	(2.90)

a 1=no dose threshold for bladder; 2=bladder threshold of 4 Gy; 3=dose to the bladder not included.

### H. Collimator rotation

**Figure 5:**
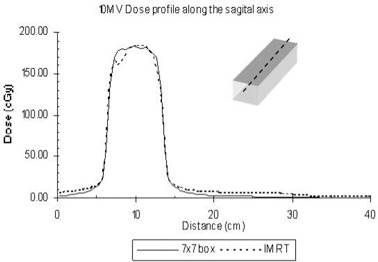
Dose profile comparison along the superior–inferior patient axis for delivery by intensity‐modulated radiation therapy (IMRT) and by four‐beam box (FBB) using 7×7−cm fields.

The collimator rotation affects the whole‐body dose equivalent and hence the percentage likelihood for secondary cancers, especially because the transmission for the MLC leaves is higher than that for the jaws. If the collimator is rotated in such way that the axis of MLC movement is parallel to the patient inferior–superior axis, then the whole‐body dose is higher. This phenomenon is more profound for the IMRT cases than for the FBB ones, because the leakage through the MLC is higher because of the higher number of MUs needed for IMRT (Fig. 5, Table 3).

**Table 3:** Whole‐body dose equivalent (cGy) and estimated percentage (%) likelihood for secondary cancer from a total of 72 Gy using the four‐beam box (FBB) technique and intensity‐modulated radiation therapy (IMRT) with a 90‐degree collimator angle

	6 MV		10 MV		18 MV	
Method^a^	FBB	IMRT	FBB	IMRT	FBB	IMRT
1	148	190	177	226	201	233
	(2.96)	(3.79)	(3.54)	(4.52)	(4.01)	(4.65)
2	82	148	88	176	89	177
	(1.63)	(2.95)	(1.76)	(3.53)	(1.78)	(3.54)
3	64	138	82	168	82	174
	(1.28)	(2.75)	(1.63)	(3.35)	(1.63)	(3.47)

a 1=no dose threshold for bladder; 2=bladder threshold of 4Gy; 3=dose to the bladder not included.

### I. Modulation scaling factor

**Figure 6:**
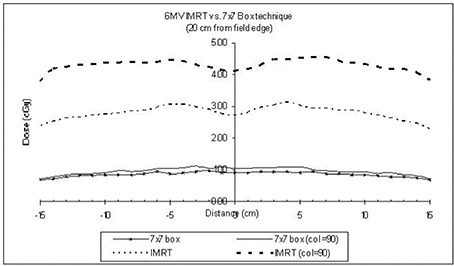
Dose profile comparison for collimator settings of 0 degrees and 90 degrees for intensity‐modulated radiation therapy (IMRT) and 7×7−cm four‐beam box (FBB) technique at 20 cm from the isocenter plane.

Comparing the MUs required for the IMRT and FBB calculations, we computed the MU factor to be 3.4. As can be seen from the Monte Carlo simulations, the overall increase in whole‐body dose equivalent is not exactly proportional to the MSF (Fig. 6). The total increase is less than tripled when the dose to the bladder is not included in the calculations or is limited to 4 Gy.

The increase observed in the whole‐body dose equivalent is approximately 0.30 – 0.40 cGy for all energies simulated. This difference can be attributed to leakage radiation through the MLC.

## IV. DISCUSSION

Estimation of the risk of secondary malignancies attributable to radiation therapy treatments is a challenging task that becomes more complex when applied in IMRT. Prostate is the most common treatment site for IMRT, and it is also usually the first site chosen for IMRT implementation in a radiation therapy department.

Our results have shown that the excess in MUs required to deliver IMRT should be considered, because that increase is associated with an increase in the risk of secondary cancers. The excessive MUs will result in increased leakage radiation that will be received by the patient's body. The increase in MUs will also depend on the plan complexity and the level of intensity modulation that is chosen to deliver the prescribed dose. The higher the modulation, the higher the leakage from the LINAC head.

The whole‐body dose is also related to the size of the fields used. For prostate treatments, field sizes are usually 7×7–10×10 cm. If the complexity of the plan is increased because proximal and distal seminal vesicles are included the overall field size, the number of MUs will also increase. That increase will also lead to an increased risk of secondary malignancies.

The choice of energy also plays an important role, because lower energies have been shown to result in lower risk for second malignancies. For departments with a choice of energies, 10‐MV photon beams are preferable, if available. The reasons are that 6 MV has insufficient penetrating power, especially for larger patients, and that 18‐MV photon beams will introduce neutrons. In our study, the neutron dose was not considered in our calculations for the equivalent effective dose, because the nominal 18‐MV photons are generated by a 14 MeV electron beam on a Siemens Primus LINAC, and we previously measured the neutron dose and found it to be insignificant.^(^ ^19^ ^,^ ^32^ ^)^ Also, in our department, prostate IMRT is generally treated with 10‐MV beams; only very rarely—for larger and older (>65 years) patients—is the 18‐MV photon energy used. For departments with Varian 18‐MV beams, the neutron dose should be estimated and included in the calculations of whole‐body dose equivalent. Based on previously published data,^(32)^ we can estimate the whole‐body dose equivalent resulting from neutrons to be approximately 2 – 5 mSv if a higher energy were to be used with the same number of MUs. This neutron dose should have been included in the calculation of the risk of second cancers, and it would have increased the risk by approximately 4% – 10%, based on the treatment modality and on the MSF.

The choice of collimator rotation was investigated, because the dose attributable to transmission through the MLC increases the whole‐body dose. Between two equivalent plans, the plan with the collimator rotated in such way that the MLC leaves are along the axial plane of the patient should be chosen so as to minimize the leakage dose to the patient. For cases in which optimal plans are not achieved with collimator orientation of this sort, jaws should be used to reduce the leakage from the MLC. This approach should be considered when radiation therapy departments are beginning to implement an IMRT program.

Moreover, our study uses 2% per gray to determine risk. Clearly, the recommended 5% per gray recommended by the International Commission on Radiation Units and Measurements (ICRU) report^(21)^ should not be applied, because it refers to a larger population spanning all ages. For the treatment of prostate cancer, the population being considered consists of men of an older age. A more conservative risk should be applied. The choice of such risk should be determined from follow‐up of men undergoing prostate IMRT. A lower percentage may perhaps be more appropriate to allow for better determination of risks of second malignancies. However, because the follow‐up data from IMRT treatments are not yet available, we feel that the 2% per gray risk used here is reasonable. Furthermore, we aimed here to show the relative increase in risk attributable to IMRT. The values used to estimate risk are mainly for study purposes, to provide readers with information about what should be expected from prostate IMRT treatments.

## V. CONCLUSIONS

Application of the IMRT technique to prostate patients has been proven beneficial with regard to reducing normal‐tissue complications while conformal dose is delivered to the target. The reduction in normal‐tissue complications allows for dose escalation to 76 Gy or higher. The drawback of IMRT is that the number of MUs required to deliver the prescribed dose is increased with respect to conventional treatments or 3D‐CRT, leading to higher doses to the patient's body as a result of leakage radiation from the LINAC head.

The relationship of field size to whole‐body dose is strong because of the fact that, for larger field sizes, the volume irradiated is larger, and so is the scatter contribution from the treated volume to organs at risk. In the case of IMRT, the irradiated volume, and not the individual segments, should be considered. The irradiated volume therefore remains the same as with conventional treatment techniques, and hence the scatter contribution is not affected.

The choice of energy has a strong effect on the equivalent effective dose. Many centers treat IMRT prostate patients with 10‐MV photon beams when available, but higher or lower energies have also been used. In the case of higher energies, the neutron dose should be included in the calculation of the whole‐body dose, because it can contribute an increase of 4% – 10% to the risk for secondary cancer. If lower energies are used, more MUs are required because of the lesser penetrating power of these beams; hence, radiation leakage is increased. In such cases, the LINAC head shielding and MLC transmission should be evaluated. Collimator rotations should be considered so as to reduce the dose to the patient.

The estimated increase in the risk of developing secondary malignancies is not proportional to the increase in MUs. For a typical IMRT prostate treatment, the MUs increase by a factor of 2 – 4, but the overall increase in the risk is about double.

In the present study, we used a 2% per gray risk for the analysis of second malignancies. Although this risk estimate is already conservative as compared with the 5% per gray proposed by ICRU,^(21)^ a more conservative estimate may be considered when comparing with future patient follow‐up data. Here, we report on the relative increase in risk attributable to the use of IMRT, rather than on prediction of the absolute risk of secondary malignancies.
